# Mothers Matter Too: Benefits of Temperature Oviposition Preferences in Newts

**DOI:** 10.1371/journal.pone.0023842

**Published:** 2011-08-24

**Authors:** Vendula Kurdíková, Radovan Smolinský, Lumír Gvoždík

**Affiliations:** Department of Population Biology, Institute of Vertebrate Biology, Academy of Sciences of the Czech Republic, Brno, Czech Republic; University of Plymouth, United Kingdom

## Abstract

The maternal manipulation hypothesis states that ectothermic females modify thermal conditions during embryonic development to benefit their offspring (anticipatory maternal effect). However, the recent theory suggests that the ultimate currency of an adaptive maternal effect is female fitness that can be maximized also by decreasing mean fitness of individual offspring. We evaluated benefits of temperature oviposition preferences in Alpine newts (*Ichthyosaura* [formerly *Triturus*] *alpestris*) by comparing the thermal sensitivity of maternal and offspring traits across a range of preferred oviposition temperatures (12, 17, and 22°C) and by manipulating the egg-predation risk during oviposition in a laboratory thermal gradient (12–22°C). All traits showed varying responses to oviposition temperatures. Embryonic developmental rates increased with oviposition temperature, whereas hatchling size and swimming capacity showed the opposite pattern. Maternal oviposition and egg-predation rates were highest at the intermediate temperature. In the thermal gradient, females oviposited at the same temperature despite the presence of caged egg-predators, water beetles (*Agabus bipustulatus*). We conclude that female newts prefer a particular temperature for egg-deposition to maximize their oviposition performance rather than offspring fitness. The evolution of advanced reproductive modes, such as prolonged egg-retention and viviparity, may require, among others, the transition from selfish temperature preferences for ovipositon to the anticipatory maternal effect.

## Introduction

Mothers contribute to offspring traits not only by shared genes but also by the influence of their phenotypes and the surrounding environment. For a long time, maternal effects have been viewed as beneficial if they improve the performance and survival of a mother's offspring [1]. However, the primary outcome of the maternal effect is currently considered maternal fitness [2], which can be maximized by producing high-quality offspring [3] or by reducing mean fitness of individual progeny for the sake of a larger clutch size [4]. The latter strategy is favored under the unpredictability of future conditions for hatchlings [5], [6] or if costs for maternal effects are high [7]. Hence, the production of superior offspring represents only one of the several ways of how a female may maximize her fitness in a given environment.

Many studies have demonstrated that ectothermic females oviposit in places that have suitable thermal conditions for embryonic development [7]–[9] (but see [10]), which suggests the anticipatory maternal effect of temperature oviposition preferences [2]. This type of maternal effect has been formulated into the maternal manipulation hypothesis [12]: ‘Mothers enhance fitness-relevant phenotypic traits of their offspring by manipulating thermal conditions during embryogenesis’. However, temperature oviposition preferences seem equally important for female performance, especially in species with a prolonged egg-laying period in temperate areas. Seasonal time constraints can favor high oviposition rates [13]. Oviposition rates are temperature-dependent [13], [14], and thus it seems likely that time-limited females prefer particular temperatures for oviposition primarily to maximize their egg-laying performance.

Since the anticipatory maternal effect of oviposition preferences should minimize offspring mortality/growth ratio [15], a possible way to discriminate between the female/offspring benefits of temperature oviposition preferences is to manipulate predation risk on laid eggs at favored oviposition temperature. In the present study, we adopted this approach to examine benefits of preferred oviposition temperatures (hereinafter *T*_p,o_) in the alpine newt, *Ichthyosaura* (formerly *Triturus*) *alpestris* (Laurenti, 1768), which is a suitable model species for this purpose. Female newts show distinct temperature oviposition preferences [16]. A whole clutch consists of 200–300 eggs [17] that are laid and wrapped individually into leaves of aquatic vegetation and the individual oviposition period lasts for several weeks. All of these facts prolong the gestation period. Since carrying egg load is costly to females, as discussed in [18]–[20], they favor maximal oviposition rates. On the other hand, water temperatures at oviposition sites provide fairly predictable cues about future thermal conditions for offspring [21], which meets the key assumption of anticipatory maternal effect [2].

The aim of our study was to test the maternal manipulation hypothesis using the interaction between biotic (egg-predator) and abiotic factors (temperature) that affect the female's egg-laying decisions. Females of various amphibian taxa discriminate among oviposition sites according to the presence of predators [22]–[24]. Here, we predicted that if temperature oviposition preferences primarily benefit offspring, the presence of an egg predator at mean *T*_p,o_ will push females to oviposit at other temperatures or even interrupt egg laying (see [16]). To accomplish this task, (1) we quantified the influence of oviposition temperatures on female oviposition rates, offspring life-history and performance traits, (2) we measured thermal sensitivity of egg predation in the water beetle, *Agabus bipustulatus* (Linnaeus, 1767), to determine its overlap with the egg-laying performance of female newts, and finally (3) we examined *T*_p,o_ of female newts in the presence or absence of an egg predator.

## Material and Methods

### Ethics statement

All experimental procedures were approved by the Expert Committee for Animal Conservation of the Institute of Vertebrate Biology AS CR (research protocol no. 113/2009). The Agency for Nature Conservation and Landscape Protection of the Czech Republic issued permission to capture the newts (1154/ZV/2008).

### Study species and maintenance

*Ichthyosaura alpestris* is a medium-sized semiaquatic newt (total length up to 12 cm) distributed across continental Europe [25]. Newts from the central European populations typically breed from April until June. Larvae metamorphose during summer but they sometimes overwinter and finish metamorphosis during the next season. Adults feed on various invertebrates, mostly chironomid larvae. The diet of newt larvae predominantly consists of plankton crustaceans. *Agabus bipustulatus* is a small dytiscid beetle (9–11 mm) widely distributed across the Palearctic. In our study population, it commonly occurs in temporary pools used by newts for reproduction.

**Figure 1:**
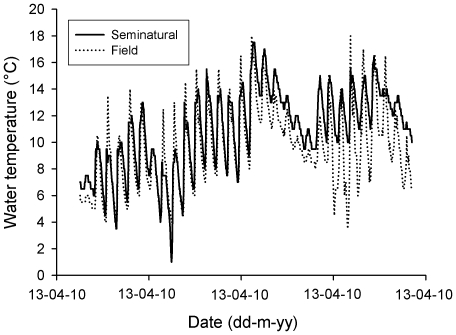
Experimental thermal conditions. Water temperatures (hourly values) experienced by newts (*Ichthyosaura alpestris*) and water beetles (*Agabus bipustulatus*) during the course of experiments in comparison with temperatures in their natural habitat.

Reproductive female newts (mean snout to vent length  = 48.3±0.7 mm; *n* = 45) and water beetles (*n* = 45) were captured from a population near Jihlava, Czech Republic, in April 2009 (female newts for plasticity experiments) and 2010 (newts and beetles for oviposition preferences). Groups of newts (two females) or beetles (five individuals) were caged in aquaria (50×30×18 cm high) filled with 15 l of tap water. Each cage was equipped with aquatic plants, *Egeria densa* and *Vesicularia dubyana*, and a piece of Styrofoam. Cages were placed outdoors in a shaded location where both species experienced natural photoperiod and temperature variations (mean±SD  = 10.9±3.0°C; see Figure 1). Newts and beetles were fed with live chironomid larvae and *Tubifex* worms twice per week. After experiments, all individuals were released at the site of their capture.

### Oviposition performance and egg incubation

Given the low sample of newt females, we applied a repeated-measures design to estimate the thermal sensitivity of oviposition performance. We placed individually reproductive females (*n* = 13) into aquaria (40×26×18 cm) with an equal amount (3 g of wet mass) of *V*. *dubyana*. Each aquarium was filled with 5 l of tap water and it was randomly distributed among three environmental rooms that maintained aquarium water temperatures at 12, 17, and 22±0.5°C and an equal 12 hour light:dark regime. Chosen water temperatures represented the mean and range of *T*_p,o_ in the study population [16]. We randomly circulated aquaria among three rooms at 36 hrs intervals. Since water temperature required several hours to equilibrate with the room temperature, eggs laid by a female during the first 12 hours were discarded from further analyses. The number of eggs laid by a female during the following 24 hrs was used as the estimate of oviposition performance at a given temperature. After oviposition performance trials, aquaria randomly circulated among rooms until each female laid the required number of eggs (*n*_eggs_ = 45) for plasticity experiments. We used eggs only from females (*n* = 8) that produced the full number of eggs during two weeks. Females were fed *ad libitum* with *Tubifex* worms, and water was replaced at three-day intervals.

We equally divided re-wrapped eggs from the each female/temperature combination into three plastic bowls (i.e. nine bowls per female). Each bowl was filled with 0.5 l of tap water, provided with an aeration stone to prevent creation of hypoxic conditions, and randomly placed into one of nine water baths (60×46×15 cm). Each bath was equipped with a 50 W aquarium heater connected to a regulation thermometer (Vertex 280, Exatherm, Jablonec n. N., Czech Republic), and a mini pump to enable equal heat distribution throughout the tank. Water baths were evenly distributed into three air-conditioned rooms with air temperatures set to maintain equilibrium water temperatures at 9, 14, and 19±2.0°C. A max-min thermometer was placed in each room to assure that the target temperature did not deviate unpredictably during incubation, e.g. in the case of a power failure. Under these conditions, aquarium heaters maintained the water bath temperatures at 12, 17, and 22±0.1°C. In this experiment, we intentionally chose constant temperature regimes to evaluate the influence of a particular oviposition temperature on offspring phenotypes. Water baths were checked everyday for the presence of dead eggs and newly hatched larvae. Evaporated water was carefully replaced (i.e. to not whirl the eggs) with deionised water during incubation.

### Swimming performance trials

We measured maximal swimming speed in a randomly selected subset of hatched larvae (i.e. 199 out of 340). We placed them individually in Petri dishes (20 ml of water) in three environmental walk-in rooms, maintaining water temperatures at 12, 17, and 22±0.5°C. When water temperature in the Petri dishes equilibrated with experimental temperature a larva was placed in the middle of squared arena (30×30×5 cm) filled with water up to 1 cm. The arena was illuminated through the semi-transparent bottom to obtain accurate outlines of experimental animals. Individual larvae were stimulated to move by the gentle touch of their tail tips using a fine stainless steel probe. Each larva was provoked four times at one temperature only to obtain a mean thermal performance curve for individuals developed under a particular temperature. Swimming bouts were recorded using a digital camera (frame frequency 50 Hz; Panasonic NV-GS500, Matsushita Electric Industrial, Osaka, Japan) mounted perpendicularly above the arena. After swimming trials, larvae were digitally photographed from a dorsal view. The total length of larvae, i.e., from the tip of snout to the tip of tail, was later measured from the digital images recorded using the tpsDIG software (F. J. Rohlf, Stony Brook Univ., Stony Brook, NY, USA), and then used as a proxy for hatchling size.

Video records were processed using motion analysis software (MaxTraq, Innovision Systems, Columbiaville, MI, USA). The maximal distance travelled by each larva during 0.02 s was used for the calculation of maximum swimming speed. Each swimming trial was subjectively judged as good or bad. Bad trials (<2% per experimental group), e.g. swimming along the walls of arena, were discarded from further analyses.

### Egg predation performance

Beetles starved for three days before predation trials. Randomly chosen beetles were individually placed in aquaria (40×26×18 cm) filled with 5 l of tap water. Aquaria (*n* = 15) were equally distributed among three environmental rooms that maintained aquarium water temperatures at 12, 17, and 22±0.5°C and an equal 12 hour light:dark regime. After 12 hrs, ten newt eggs were added to each aquarium and beetles left undisturbed for the next 24 hrs. The number of consumed eggs per 24 hrs was used as an estimate of egg predation rate. To examine whether egg-wrapping into vegetation affects predation rates in this species [26], [27], we repeated trials at 17°C with wrapped and unwrapped eggs. Each beetle was used for one trial only.

### Temperature oviposition preferences

**Figure 2:**
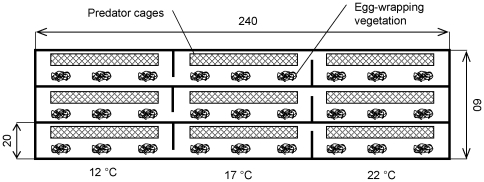
Experimental tank for testing oviposition preferences. Schematic representation of the experimental tank with three water temperatures, clumps of aquatic vegetation, and predator cages for testing the influence of water temperature and predator cues on oviposition preferences in female newts. Please note that egg-predators (aquatic beetles) were randomly placed in to cages held at 17°C.

Oviposition preferences were studied in a stainless steel tank longitudinally divided into three lanes (Figure 2). Each lane was filled with water (5 cm) and partially divided into three compartments. Using build-in Peltier modules connected to a computer unit, each compartment maintained water temperatures of 12, 17, and 22°C in each lane. Each compartment was equipped with three clumps of vegetation (*V*. *dubyana*; 3 g wet mass) and a cylindrical mesh cage (60×8 cm).

Twelve hours before experiments (20:00), five beetles were placed in the cage of randomly selected lanes at 17°C (treatment). The remaining lanes represented control. Three randomly chosen females were added individually to each lane. The number of deposited eggs day^−1^ at each temperature was recorded for a female. The tank and its equipment were thoroughly washed with ethanol, and water and vegetation was changed after each trial.

### Statistical analyses

Data were analyzed using various models according to the type of data and the design of a particular experiment. Developmental rates (i.e. the proportion of development occurring per units of time [days^−1^]), hatchling size (mm) and swimming speed (cm s^−1^) met assumptions of normality and homogeneity of variances, and thus their thermal dependence was analyzed using the general linear mixed-effect (GLMM) model [28]. Since the developmental temperature (*T*_dev_) was considered an ordinal categorical variable, orthogonal polynomial contrasts were used for this model. The polynomial constrasts of *T*_dev_ (i.e. linear [L] and quadratic [Q]) represented fixed factors, whereas water baths nested within *T*_dev_ and family identity, were added as random factors. To analyse swimming speed, the GLMM included additional two fixed factors–experimental temperature (*T*_exp_; including interaction with *T*_dev_), and hatchling total length as the covariate.

Numbers of eggs laid are counts, and thus we used the generalized linear mixed-effect model (GLZMM) for Poisson-distributed data (log-link function) to test the thermal sensitivity of oviposition performance. In case of overdispersion, a “quasi-likelihood” model (quasi-Poisson) was applied. The model consisted of two fixed factors—linear (L) and quadratic (Q) contrasts of *T*_exp_ and a random grouping factor–female identity.

The thermal dependence of embryonic survival was examined with GLZMM for binomially-distributed data (logit link function). Newt oviposition rates in the thermal gradient were analyzed using GLZMM with fixed factors, i.e. predator presence, oviposition temperature, and their interaction, and female identity as a random grouping factor.

A significance level of α  = 0.05 was used for all statistical tests. The statistical significance of random factors was tested using the likelihood ratio approach [29]. All means are reported ± one standard error. Analyses were performed using R (library nlme and lme4) statistical packages (R Foundation for Statistical Computing, Vienna, Austria).

## Results

**Figure 3:**
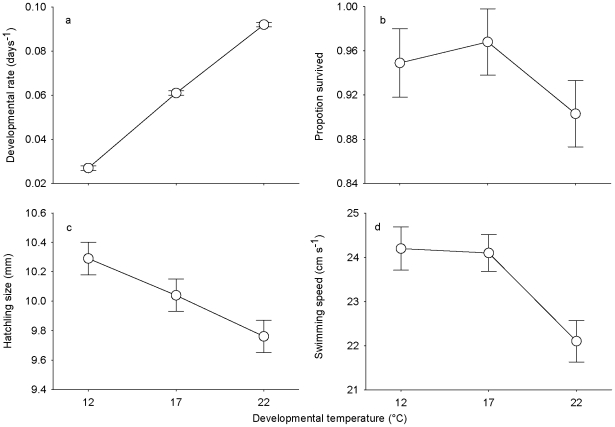
Thermally-induced plasticity of offspring traits. Embryonic (a) developmental and (b) survival rates, (c) hatchling size, and (d) swimming speed of freshly hatched larvae that developed under three preferred oviposition temperatures of female newts (*I. alpestris*). Swimming speed represents size-adjusted means across three experimental temperatures (12, 17, and 22°C). Values refer to mean±SE.

Temperatures experienced during embryonic development affected both embryonic and hatchling traits (Figure 3). While developmental rates sharply increased with developmental temperature (*T*_dev_[L]: *t*_6_ = 41.33, *P*<0.001), hatchling size decreased (*T*_dev_[L]: *t*_6_ = 41.33, *P*<0.001). The influence of *T*_dev_ on embryonic survival was statistically non-significant (*T*_dev_[L]: *z* = 1.30, *P* = 0.20; *T*_dev_[Q]: *z* = 1.37, *P* = 0.17). Swimming speed of hatchlings increased with *T*_exp_ (*F*_188_ = 67.26, *P* = 0.77) at similar rates despite their development at various temperatures (*T*_dev_ × *T*_exp_: *F*_188_ = 0.26, *P* = 0.77). The mean swimming speed across all *T*_exp_ decreased with *T*_dev_ (*T*_dev_[L]: *t*_6_ = 3.24, *P* = 0.016), because it was markedly lower in larvae developed at 22°C than in lower temperatures.

**Figure 4:**
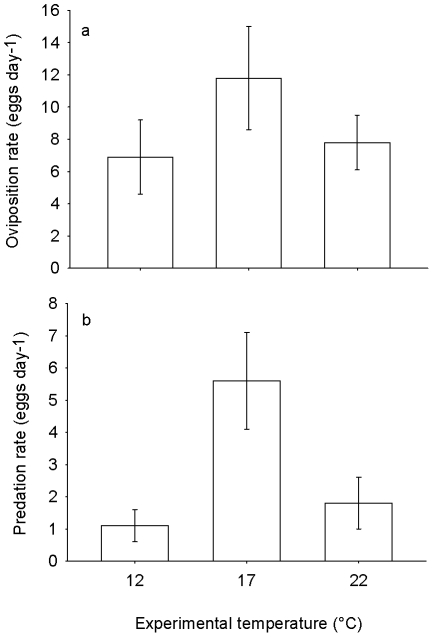
Thermal sensitivity of oviposition and egg predation. (a) oviposition rates in newts (*I. alpestris*) and (b) egg-predation rates by water beetles (*A. bipustulatus*) at three experimental temperatures. Values refer to means±SE.

**Figure 5:**
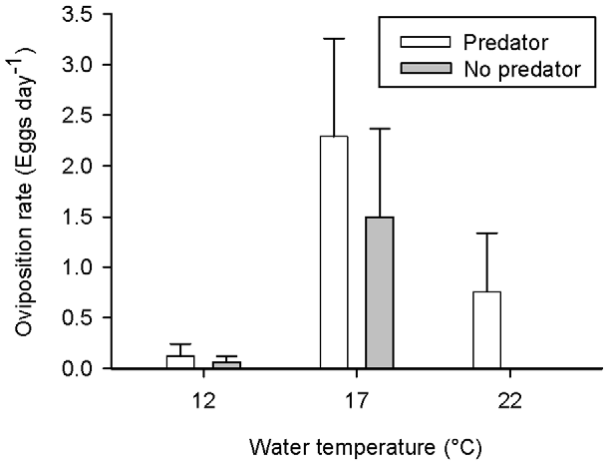
Oviposition in thermal gradient. Oviposition rates of female newts (*I. alpestris*) under thermal gradient and in the presence/absence of a caged egg-predator (*A. bipustulatus*) at 17°C. Values refer to means+SE.

Water beetles consumed wrapped and unwrapped newt eggs at similar rates (4±1 vs. 6±1 eggs day^−1^, respectively; *t*_17_ = 0.92, *P* = 0.37). Predation rates of unwrapped eggs were higher at 17°C than at adjacent temperatures (*T*_exp_[Q]; *z* = 3.74, *P*<0.001; Figure 4a). The thermal sensitivity of newt oviposition rates showed the same pattern both in oviposition performance trials and in the thermal gradient (trials: *t*_26_ *= *3.27, *P = *0.003; gradient: *t*_62_ *= *2.16, *P = *0.03; Figure 4b, 5). Females oviposited at similar rates irrespective of the presence of caged water beetles (*t*_31_ = 1.21, *P* = 0.23; Figure 5), which suggests that their oviposition decisions were unaffected by the egg-predation risk.

## Discussion

The current theory implicitly predicts that an ovipositing female may maximize her fitness by the selection of oviposition sites where conditions benefit offspring performance, her oviposition performance, or both [2]. In case of temperature oviposition preferences, the prevailing view considers only the first option, i.e. the anticipatory maternal effect [12], [10], (but see [11]). Our results showed that mean *T*_p,o_ indeed provided the best compromise for disparate thermal reaction norms of offspring traits, which seems congruent with the maternal manipulation hypothesis. Whilst egg-predation risk does not discourage females from oviposition at the same temperature, it does increase the mortality risk/growth ratio of her offspring. Since the mean *T*_p,o_ also maximizes oviposition rates, our results suggest that temperature oviposition preferences primarily mediate ‘selfish’ rather than ‘anticipatory’ maternal effect in newts.

Besides selfish oviposition preferences, it may be argued that the insensitivity of *T*_p,o_ to egg-predation risk resulted from at least two other factors. First, one might assume that temperature oviposition preferences mediated an anticipatory maternal effect but temperature was a more important factor determining offspring fitness than predation risk. Our results disagree with this possibility. Although *T*_dev_ influences offspring phenotypes (Figure 3), development at temperatures lower than 17°C (i.e. the temperature with the highest risk of predation) proceeded without reduced embryonic survival and swimming capacity of hatched larvae.

Second, one might assume that egg-predator avoidance is an ineffective oviposition strategy in a natural newt habitat. Theory predicts that two characteristics of our model system, i.e. spatiotemporal spread of laid eggs and uniform predator distribution between potential oviposition sites, should disfavour the evolution of oviposition site preferences in response to egg predators [30]. However, these predictions pertain to microhabitat preferences (i.e. among pools) rather than to microsite oviposition-site choice (i.e. within the pool). In our case, deeper water bodies used by newts for reproduction create a vertical thermal gradient during the daytime [21] and egg predation by water beetles is thermally sensitive (Figure 4a). Contrary to the proposed explanation, our study proves female newts have indeed the potential to reduce predation risk by oviposition at temperatures suboptimal for egg predators.

If the selfish character of temperature oviposition preference holds, a good match between mean *T*_p,o_ and ‘thermal compromise’ among disparate thermal reaction norms for offspring traits arose for reasons other than a mother's effort to provide the best conditions for embryonic development. The likely explanation seems the common selection pressure of a thermal environment on a mother and its offspring. The selection acting on thermal biology traits should be the most intense at the modal temperature [31]. Congruently, mean oviposition preferences and the ‘thermal compromise’ of offspring reaction norms are close to modal temperatures at their natural oviposition sites [21], which suggests a good fit of both maternal and offspring phenotypes with their local adaptive optima.

Theory predicts that this situation leads either to the evolution of a mother-offspring genetic correlation [32] or to phenotypic plasticity of female oviposition preferences or offspring performance [33]. The minimal variation in *T*_p,o_ of female newts exposed to different thermal regimes ([16] vs. this study) in comparison with the pronounced developmental plasticity of offspring traits ([34], this study) clearly corroborates the latter scenario in our model species. Hence, it seems likely that female newts primarily preferred the best conditions for egg-laying performance, which was followed by the evolution of thermal reaction norms in their offspring.

Although the primary benefit of *T*_p,o_ in newts seems maternal oviposition performance, the match between this trait and the thermal compromise of disparate reaction norms for offspring traits (Figure 3) suggests that under the absence of egg-predators, maternal oviposition choice provides the opportunity for the anticipatory maternal effect. In our study population, we detected water beetles in most pools used by newts for reproduction (J. Dvořák, R. Smolinský, and L. Gvoždík, unpublished observations). Hence, depending on egg-predator densities, maternal effect of *T*_p,o_ may vary within the ‘selfish–anticipatory’ continuum in the field. Given the context-dependency of *T*_p,o_, benefits of this behavioral maternal effect should be always considered with caution (see also [2]).

In conclusion, our study revealed the primarily selfish character of temperature oviposition preferences in alpine newts. The results have two important implications. First, time limited females carefully choose oviposition temperatures (i.e. spatially and/or temporally) with the aim of disposing an egg load as fast as possible (see also [35]). The oviposition theory predicts that time-limited females with a high egg load should be less picky in their choice of oviposition sites than females with few eggs and plenty of time [36]–[38]. If oviposition rates are temperature-dependent, this prediction seems reasonable for choice of a suitable host in parasitic species although we propose exactly the opposite for temperature oviposition preferences. Secondly, alpine newts were found to exhibit selfish oviposition preferences, which belong to species with an ancestral reproductive mode, i.e. oviposition at the beginning of embryogenesis, whereas the anticipatory maternal effect seems widespread among taxa with advanced reproductive modes, such as prolonged egg-retention and viviparity [7]–[9]. This suggests that besides various morphological and physiological prerequisites [39], [40], the evolution of advanced reproductive modes requires another not yet considered condition – the transition of selfish temperature oviposition preferences to the anticipatory maternal effect.
